# Comparative Neuroanatomy of Hydrothermal Vent Shrimps: Ecological Differentiation Among Four Alvinocaridid Species

**DOI:** 10.1002/cne.70190

**Published:** 2026-07-28

**Authors:** Adrien Mathou, Julia Machon, Rebecca Meth, Magali Zbinden, Juliette Ravaux, Steffen Harzsch

**Affiliations:** ^1^ UMR CNRS MNHN 8067 Biologie des organismes et écosystèmes aquatiques (BOREA), Équipe Adaptation aux Milieux Extrêmes Sorbonne Université Paris France; ^2^ Department of Cytology and Evolutionary Biology, Zoological Institute and Museum University of Greifswald Greifswald Germany; ^3^ UMR CNRS MNHN 8067 Biologie des organismes et écosystèmes aquatiques (BOREA), Équipe Biodiversité, plasticité, adaptation et conservation des espèces aux communautés Sorbonne Université Paris France

**Keywords:** alvinocaridid, brain evolution, hydrothermal vents, neuroanatomy, sensory organs

## Abstract

Alvinocaridid shrimps colonize hydrothermal vent fields along the Mid‐Atlantic Ridge (MAR), where they experience extreme environmental conditions including high hydrostatic pressure, steep thermal and chemical gradients, and dim light. While various aspects of the biology of the dominant vent shrimp *Rimicaris exoculata*, such as general anatomy, microhabitat, symbiotic relationships, or trophic networks, have been extensively studied, little is known about how brain organization varies among alvinocaridid species occupying different ecological niches. Here, we investigated whether vent shrimps share common neuroanatomical features and whether differences in brain and vascular organization reflect ecological diversification among species. Using histology, immunohistochemistry, µCT scans, and 3D reconstruction, we compared the brain and the neurovascular system (*cor frontale*) in four MAR species with distinct ecologies (*R. exoculata*, *Rimicaris chacei*, *Mirocaris fortunata*, *Alvinocaris markensis*) and in the coastal relative *Palaemon elegans*. Our results reveal strong commonalities in brain organization among vent shrimps. All MAR species display a compact brain architecture with hypertrophied mushroom bodies (MBs), suggesting enhanced multisensory integration and memory capacities. Corresponding to the low‐light environment, the visual neuropils (lamina, medulla, lobula) are smaller than those of the coastal species *P. elegans*. In all species living near hydrothermal vents, the retina exhibits modifications; it extends along the dorsal part of the cephalothorax, and its length varies according to the species, being the longest in the genus *Rimicaris*. The lobula satellite neuropil is absent in all alvinocaridids, suggesting a limited capacity to track moving visual signals. The olfactory lobes exhibit a common glomerular organization but differ among species in quantitative parameters, such as volume and number of glomeruli. The neurovascular system shows marked interspecific variation; the *cor frontale* differs in size and volume among hydrothermal vent species but retains a conserved subdivision of central arteries surrounding a longitudinal muscle bundle. We conclude that the observed variations in the volume of neuropils and arrangement of the vascular system likely reflect the ecological divergence related to microhabitat conditions and trophic strategies near hydrothermal emissions.

## Graphical abstract

Hydrothermal vent shrimps share a conserved brain architecture with enlarged mushroom bodies and terminal medullae, shaped by similar environmental pressures. Despite this similarity, some anatomical features near the brain vary between species, suggesting that some ecological factors, such as microhabitat and trophic strategies, also influence nervous system adaptations in vent shrimps.

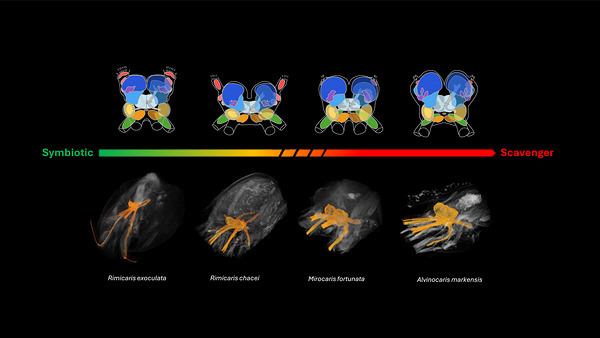

## 1 Introduction

**FIGURE 1:**
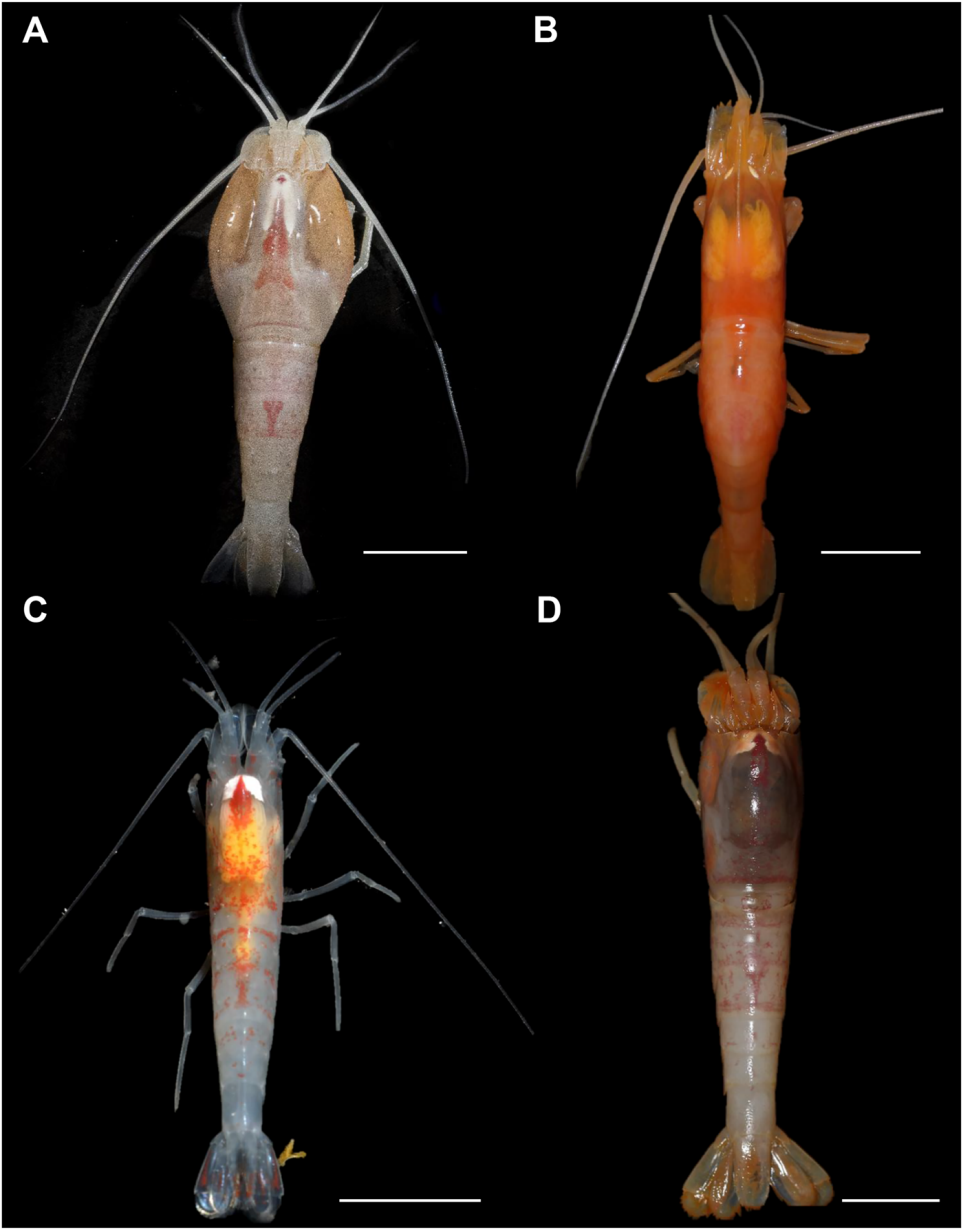
Postcapture photographs of the four alvinocaridids from MAR in dorsal view. (A) *Rimicaris exoculata* (symbiotic). (B) *Alvinocaris markensis* (scavenger) (Valérie Cueff‐Gauchard). (C) *Mirocaris fortunata* (scavenger) (Océanopolis). (D) *Rimicaris chacei* (mixotrophic) (Laure Corbari). Scale bars = 1 cm.

Hydrothermal vent fields are extreme environments distributed worldwide along oceanic ridges and other volcanic areas (Baker and German 2004). They are characterized by low light, high pressure, and steep thermal and chemical gradients, where hot anoxic fluids from chimneys mix with cold (∼4°C) oxygenated seawater. In these ecosystems, chemoautotrophic microorganisms form the trophic base by producing organic matter for primary consumers (Jannasch and Mottl 1985). Among the dominant macrofauna, alvinocaridid shrimps are the most prevalent organisms and are widely distributed along the Mid‐Atlantic Ridge (MAR) (Van Dover 1995; Gebruk and Galkin 1997; Lunina and Vereshchaka 2014). *Rimicaris exoculata* (Williams and Rona 1986; see Figure 1) is one of the most studied species due to its high abundance and its role as a primary consumer (Vereshchaka et al. 2015; Zbinden and Cambon‐Bonavita 2020). This shrimp relies on symbiotic chemoautotrophic bacteria for nutrition (Ponsard et al. 2013; Zbinden and Cambon‐Bonavita 2020). Three other Alvinocarididae species are also commonly found on MAR vent fields and can be distinguished from *R. exoculata* by their morphology, spatial distribution, and trophic ecology: *Rimicaris chacei*, *Mirocaris fortunata*, and *Alvinocaris markensis* (Figure 1). *Rimicaris chacei* lives close to vent emissions, often only centimeters from fluids and near *R. exoculata* swarms, but at lower densities (Gebruk and Galkin 1997). In contrast, *M. fortunata* and *A. markensis* (Komai and Segonzac 2005) are found several meters away from chimneys, often associated with mussel beds. *Rimicaris chacei* is mixotrophic, relying on both symbionts and external food sources (Gebruk et al. 2000), whereas *M. fortunata* and *A. markensis* are scavengers and predators, less dependent on hydrothermal fluids (Desbruyères et al. 2006). Given the steep environmental gradients, these shrimps likely use multiple sensory modalities to locate habitats and food (Charmantier‐Daures and Segonzac 1998; Jinks et al. 1998; Derby and Caprio 2024).

Vision and chemoreception are considered two major sensory modalities used by these shrimps to perceive their environment, as they possess functional sensory organs such as olfactory sensilla (aesthetascs) and a retina (Chamberlain 2000; Zbinden et al. 2017). However, convincing behavioral and neurophysiological evidence on how these senses are used and their efficiency remains limited. In hydrothermal vent shrimps, particularly *R. exoculata*, the stalked compound eyes typical of many malacostracan crustaceans are fused into an enlarged sessile eye and lack a dioptric apparatus (Chamberlain 2000; Gaten et al. 1998a, 1998b). This modification prevents image formation but, through an enlarged retina and numerous pigmented photoreceptors, likely enables detection of near‐infrared radiation (>700 nm) emitted by hydrothermal fluids (Pelli and Chamberlain 1989).

Malacostracan crustaceans possess two sets of cephalic chemoreceptive appendages. The anterior pair (antennules or antenna 1) contains both specialized unimodal olfactory sensilla (aesthetascs) and bimodal chemo‐ and mechanosensilla. In contrast, the second pair (antennae or antenna 2) is equipped solely with bimodal chemo‐ and mechanosensilla (for review, see Hallberg et al. 1992; Mellon 2007; Hallberg and Skog 2010; Derby and Weissburg 2014; Derby 2021). Alvinocaridids present lateral antennules (antenna 1) equipped with aesthetascs, suggesting a functional olfactory apparatus. The aesthetascs in alvinocaridids seem to have relatively similar dimensions to those of their coastal relative *Palaemon elegans*, but are fewer in number (Zbinden et al. 2017). The main divergence is observed for the bimodal, combined chemo‐ and mechanosensory sensilla, which display a variety of morphologies depending on the species observed (Zbinden et al. 2017). Machon et al. (2018) showed that the elements of the peripheral chemosensory pathway of alvinocaridids are not sensitive to long‐range hydrothermal stimuli such as iron or manganese, whereas short‐range stimuli such as sulfide or food‐related odors are detected by both antennules (antenna 1) and antennae (antenna 2). However, these short‐range stimuli can also be detected by the coastal species, *P. elegans* (Machon et al. 2018). Besides the chemical senses, thermal detection is another important sensory modality in crustaceans, although the neuronal basis of this sense remains unclear. Temperature strongly influences the distribution of vent fauna around active chimneys (Sarrazin et al. 1997; Lee 2003) and may act as a directional cue. Indeed, *R. exoculata* and *M. fortunata* have been shown to be attracted to water at 11°C (Ravaux et al. 2009; F. Smith et al. 2013; Ravaux et al. 2021).

In vent shrimps, environmental stimuli related to, for example, temperature, light, and odors are supposedly used to navigate within vent fields and to locate and remember habitats and food sources. Such stimuli are processed by the central nervous system, mainly the brain (cerebrum), to generate adequate physiological or behavioral responses. The architecture of the brain, specifically of the synapse‐dense regions called neuropils, has been thoroughly analyzed in a variety of decapod crustaceans (reviewed in D. Sandeman et al. 1992; D. C. Sandeman et al. 2014; Loesel et al. 2013; Schmidt 2015; Strausfeld 2018). The input from the (bilaterally paired) compound eyes is processed in the visual neuropils: lamina, medulla, lobula, and the lobula satellite neuropil, all of which are located within the eyestalks. The terminal medulla (TM) and mushroom bodies (MBs), neuropils in the (bilaterally paired) eyestalks, act as higher‐order multimodal centers integrating diverse sensory input. The input from sensory neurons associated with the aesthetascs and bimodal sensilla on the (bilaterally paired) antennules (antenna 1) is processed in the (bilaterally paired) olfactory lobes (OLs) and the lateral antenna 1 neuropils (LANs) (D. Sandeman et al. 1992; D. C. Sandeman et al. 1993; reviewed in, e.g., Schmidt and Mellon 2011; Derby and Weissburg 2014; D. C. Sandeman et al. 2014; Harzsch and Krieger 2018). The OLs provide an input to the MBs via the projection neuron tracts (PNTs). Sensory inputs from the (bilaterally paired) antennae (antenna 2) target the antenna 2 neuropils (A2Ns) (D. Sandeman et al. 1992; D. C. Sandeman et al. 1993; reviewed in Schmidt 2015).

To match the high energy demands of nervous tissue, the crustacean brain is closely associated with elements of the circulatory system, including, for example, the ophthalmic arteries (OAs) and central cerebral arteries (Schmidt 2015). The anterior aorta directs hemolymph from the heart directly to the brain (Wirkner and Richter 2013; Davie et al. 2015). The *cor frontale* is a myoarterial formation associated with the anterior aorta present in many adult decapod crustaceans (Steinacker 1978). This vascular structure in crustaceans represents an aortic dilation with its own intrinsic muscles. It acts as an auxiliary heart to provide a stable hemolymph flow from the heart to the central brain and the eyestalk neuropils (see Wirkner and Richter 2013). Machon et al. (2019) had noticed that *R. exoculata* showed a singular morphology in this myoarterial formation, questioning the efficiency of this associated organ in the maintenance of a stable energy supply for the different brain domains.

Ecological shifts are often accompanied by morphoanatomical changes affecting the structure of specific organs or body regions (deVries 2017; Weihmann 2020; Lin et al. 2021; Marin and Tiunov 2023). In alvinocaridids, Methou et al. (2024) described such differences among *R. exoculata*, *R. chacei*, and *Rimicaris hybisae*, including modifications of the visual system and changes in gill chamber morphology associated with symbiont colonization. More generally, comparative studies in crustaceans have suggested that the relative investment in nervous tissues of particular brain regions reflects the animal's sensory ecology (reviewed in D. C. Sandeman et al. 2014). For instance, cavernicolous peracarid and remiped crustaceans lack visual neuropils but possess well‐developed OLs, suggesting a strong reliance on olfaction for environmental perception (Fanenbruck et al. 2004; Fanenbruck and Harzsch 2005; Stegner et al. 2015; Stemme and Harzsch 2015). Sensory organs and their associated brain centers can also evolve in response to ecological transitions. During terrestrialization, several crustacean groups including talitroids, woodlice (Isopoda), and crabs (Brachyura) show reductions in the size of the antennules (antenna 1), aesthetasc sensilla, and primary olfactory processing centers compared to their aquatic relatives (Krieger et al. 2015; Harzsch and Krieger 2018; Krieger et al. 2020; Marin and Tiunov 2023), highlighting the strong influence of ecology on brain organization.

Although hydrothermal vent shrimps share similar environmental conditions (dim light, high hydrostatic pressure, etc.), their ecological niches and trophic strategies differ markedly. *Rimicaris chacei*, *M. fortunata*, and *A. markensis* coexist with *R. exoculata* but occupy distinct microhabitats and use different feeding strategies, from symbiosis to mixotrophy or scavenging. These differences raise the question of whether brain organization and neuropil structure reflect species‐specific adaptations. What is more, we may ask how important certain sensory modalities, such as olfaction, are for hydrothermal crustacean behavior compared to other senses, such as thermal detection or vision. To address these questions, we examined and compared the structure of selected sensory organs (eyes, antennules/antenna 1, and organ of Bellonci [OB]), the brain, and aspects of the brain hemolymph supply by the auxiliary heart in *R. chacei*, *M. fortunata*, and *A. markensis* with that of *R. exoculata* (previously described by Machon et al. 2019) and their shallow‐water relative, *P. elegans*. By targeting closely related species that span a gradient of feeding strategies and microhabitat use, this study aims to test whether ecological specialization within the extreme but heterogeneous hydrothermal environment drives measurable neuroanatomical divergence.

## 2 Materials and Methods

### 2.1 Specimen Sampling and Fixation

Individuals of four species of shrimp belonging to the Alvinocarididae (*R. exoculata*, *R. chacei*, *A. markensis*, *M. fortunata*) were collected along the MAR, during the BICOSE 2 oceanographic expedition in January 2018 on board the research vessel “Pourquoi pas?” (Figure 1; Table 1). Sampling took place at two sites: TAG (26°08ʹN–44°49ʹW, at 3600 m depth) and Snake Pit (23°22ʹN–44°57ʹW, 3450 m depth). The specimens were recovered using the submersible “Nautile 6000” and brought back on board using the “PERISCOP” pressurized transport system (Shillito et al. 2023). Once recovered, the individuals were sorted by sex. Only females were kept for observations to avoid any bias linked to potential sexual dimorphism (*n* = 3 per species). For each specimen, the cephalothorax was then isolated from the pleon, and the hepatopancreas was removed for fixation. Three specimens per species (one for confocal microscopy, one for histological observations, and one for micro‐computed X‐ray tomography) were used for this study. Specimens intended for immunohistochemistry were fixed in 4% paraformaldehyde (PFA) in seawater for 24–48 h at 4°C, briefly rinsed in 0.1 M phosphate‐buffered saline (PBS) at 4°C, and stored in 0.1 M PBS/NaN_3_ at 4°C until use. Specimens intended for micro‐computed X‐ray tomography (µCT scan) observations and histology were fixed and conserved in Bouin's solution (10% formaldehyde, 5% glacial acetic acid in saturated aqueous picric acid) at 4°C until use.

**TABLE 1:** Ecological description of alvinocaridid shrimps from the Mid‐Atlantic Ridge (Gebruk and Galkin 1997; Gebruk et al. 2000; Desbruyères et al. 2006; Cuvelier et al. 2009, 2011; Bates et al. 2010; Lunina and Vereshchaka 2014; Sarrazin et al. 2015).

Species	Depth range	Microscale distribution	Temperature range	Trophic mode	Population density
*Rimicaris exoculata*	2270–4080 m	Vicinity of active chimneys, shimmering water	4°C–40°C	Primary consumer	2500 ind/m^2^
*Rimicaris chacei*	1600–4080 m	Vicinity of active chimneys, shimmering water, mussel beds	4°C–14°C	Primary and secondary consumer (mixotrophic)	50–200 ind/m^2^
*Mirocaris fortunata*	840–4080 m	Mussel beds, vent periphery	4°C–14°C	Secondary consumer	<10 ind/m^2^
*Alvinocaris markensis*	1600–3670 m	Mussel beds, vent periphery	4°C–14°C	Secondary consumer	30 ind/m^2^
*Palaemon elegans*	0.2–7.8 m	Rocky foreshore	8°C–30°C	Secondary consumer	50–100 ind/m^2^

### 2.2 Histology

The cephalothorax of four animals (one per species) fixed in Bouin's solution were subjected to dehydration through a graded ethanol series. Samples were dehydrated at room temperature on a shaker through a graded ethanol series: 80%, 90%, and 95% (twice, 30 min each), followed by two baths of 99% ethanol (60 min, then overnight). Samples are then embedded in paraffin containing 5% beeswax. Serial sections, 7‐µm thick, were cut in either the frontal or sagittal plane using a Leica RM 2145 microtome (Leica Microsystems, Wetzlar, Germany). The tissue sections were stained with Azan‐novum following the method described by Geidies (1954), in accordance with standard protocols outlined by Welsch and Mulisch (2010).

### 2.3 Immunohistochemistry and Sample Staining

The brains of the various specimens fixed in 4% PFA solution were rinsed and dissected in PBS saline (0.1 M, pH 7.4), then coated in poly‐I‐lysine for 1 min and embedded and solidified in low‐gelling agarose for 2 min at room temperature (8% agarose: 1.2 g agarose in 15 mL ddH_2_O, dissolved in microwave) (Cat. A9414; Sigma–Aldrich Chemie GmbH, Munich, Germany). The resulting agarose block was cut using a vibratome (Hyrax V50). The sections (100‐µm thick) were then incubated in a solution of PBT (PBS with 0.3% Triton X‐100 and 1% bovine serum albumin) for 1.5 h to facilitate antibody penetration. Two combinations of antisera for the brain sections were used in the following. The first set included an anti‐synapsin “SYNORF1” antiserum (DSHB, 3C11; mouse; dilution 1:10; RRID: AB_2313867), an anti‐allatostatin “anti‐A‐allatostatin polyclonal” antiserum (A‐type Dip‐allatostatin I; Jena Bioscience, abd‐062; rabbit; dilution 1:1000; RRID: AB_2314318), and a nuclear marker. After 2 days of incubation at room temperature, samples were repeatedly rinsed in PBT for 1 h (2 × 10 min and 2 × 20 min). Samples are then incubated overnight at room temperature with secondary antisera (anti‐IgG) coupled to fluorescent markers: Fluor 488 (Alexa Fluor 488 goat anti‐rabbit IgG Antibody, Invitrogen, Thermo Fisher Scientific, Waltham, MA, USA; dilution 1:500; RRID: AB_10374301) and Cy3 (Cy3‐conjugated AffiniPure Goat Anti‐Mouse IgG Antibody, Jackson ImmunoResearch Laboratories Inc., West Grove, PA, USA; dilution 1:500; RRID: AB_2338000). The complementary nuclear marker HOECHST (Cat. 14530; Sigma–Aldrich Chemie GmbH) was used to visualize neuron nuclei. Finally, sections were washed several times in PBT for 2 h (2 × 10 min, 2 × 20 min, and 1 h), then mounted with Mowiol 4–88.

### 2.4 Antibody Specificity

#### 2.4.1 Synapsin

The monoclonal anti‐SYNORF1 synapsin antibody (DSHB Hybridoma Product 3C11; anti SYNORF1 as deposited to the DSHB by E. Buchner) was raised against a *Drosophila melanogaster* GST‐synapsin fusion protein and recognizes at least four synapsin isoforms (70, 74, 80, and 143 kDa) in western blots of *D. melanogaster* head homogenates (Klagges et al. 1996). Sullivan et al. (2007) showed a single band at about 75 kDa in a western blot analysis of crayfish homogenates. Harzsch and Hansson (2008) conducted a western blot analysis comparing brain tissue of *D. melanogaster* and the hermit crab *Coenobita clypeatus* (Anomura, Coenobitidae). The SYNORF1 serum provided identical results for both species, and it stained one strong band between 80 and 90 kDa and a second weaker band slightly above 148 kDa, suggesting that the epitope that SYNORF1 recognizes is strongly conserved between *D. melanogaster* and *C. clypeatus* (see Harzsch and Hansson 2008). Similar to the fruit fly, the antibody consistently labeled brain structures in other major subgroups of the malacostracan crustaceans in a pattern consistent with the assumption that this antibody labels synaptic neuropils in crustaceans (Harzsch and Glötzner 2002; Beltz et al. 2002; Krieger et al. 2012; Machon et al. 2019; Krieger et al. 2020). The antibody also labels neuromuscular synapses in Crustacea and *Drosophila* (Harzsch and Glötzner 2002).

#### 2.4.2 Allatostatin

The A‐type allatostatins represent a large family of neuropeptides that were first identified from the cockroach *Diploptera punctata*; they additionally share the C‐terminal motif ‐YXFGLamide (Stay et al. 1995; Stay and Tobe 2007; Wilson and Christie 2010). In the shore crab *Carcinus maenas* (Brachyura), almost 20 native A‐type allatostatin‐like peptides were identified from extracts of the thoracic ganglia (Duve et al. 1997). Shortly afterward, various other A‐type allatostatin‐like peptides were isolated from the Eastern Crayfish *Orconectes limosus* (Astacida; Dircksen et al. 1999). Meanwhile, A‐type allatostatin peptides have been discovered in a wide range of malacostracan crustaceans, including Brachyura (Huybrechts et al. 2003), the prawns *Penaeus monodon* (Duve et al. 2002), *Macrobrachium rosenbergii* (Yin et al. 2006), and the shrimp *Penaeus vannamei* (Meth et al. 2017). Christie and Pascual (2016) identified a total of 29 peptides with the C‐terminal motif, ‐YXFGLamide, in the latest analysis on the peptidome of the shore crab. The polyclonal rabbit allatostatin antiserum used in the present study was raised against the *Diploptera punctata* A‐type Dip‐allatostatin I, APSGAQRLYGFGLamide, coupled to bovine thyroglobulin using glutaraldehyde. It has previously been used to localize A‐type allatostatin‐like peptides in crustacean and insect nervous systems (Polanska et al. 2012). In the following, the term “allatostatin‐like immunoreactivity” is used to indicate that the antibody most likely binds to various related peptides within this peptide family. Immunohistochemistry against A‐type allatostatins is well suited for labeling specific neuron types such as the olfactory interneurons in crustaceans (Polanska et al. 2012; Harzsch and Krieger 2021). Therefore, we primarily used this marker to analyze interneurons innervating the OLs and particularly their subunits, the olfactory glomeruli. A‐type allatostatins also allow us to observe other neuronal networks innervating higher brain structures, such as the striations on A2N and LAN, as already observed in *R. exoculata* in Machon et al. (2019).

### 2.5 Imaging

Tissues prepared for immunofluorescence were first observed under a fluorescence microscope (Nikon Eclipse 90i) to assess labeling efficiency and identify areas of interest for confocal scanning. Samples were then analyzed using confocal laser scanning microscopy (Leica TCS SP5II) equipped with DPSS lasers (diode and argon) via LASAF software (Leica Microsystems). The images obtained were processed with ImageJ to adjust contrast and brightness. To ensure accessibility for color‐blind readers, markers followed Color Universal Design standards: SYN (magenta), AstA (green), and NUC (cyan) (Ichihara et al. 2008, Ichihara et al. 2010).

### 2.6 Iodine Staining, Micro‐Computed X‐Ray Tomography, and 3D Reconstruction

The cephalothorax of specimens fixed in Bouin's solution was rinsed in PBS (3–6 × 15 min). Samples were then contrasted with iodine solution (2% resublimed iodine in 99.5% ethanol) for 48 h at room temperature. After staining, samples were rinsed with absolute ethanol (2 × 15 min) and stored for critical‐point dehydration. µCT scans were taken using an “Xradia MicroXCT‐200” X‐ray microscope (Sombke et al. 2015). All specimens were critical‐point dried (Leica EM CPD300, Leica Microsystems, Wetzlar, Germany) before scanning to preserve fine structures of the central nervous system. Only *M. fortunata* was scanned in ethanol in order to ensure optimal contrast (e.g., stabilization of iodine, passive contrast achieved through differential tissue dehydration, emphasizing differences in tissue density), given that *M. fortunata* has a smaller brain than other species, making X‐ray tomography scans more difficult for these individuals using the available equipment. The images obtained by µCT are reconstructed via the “XMReconstructor” software, generating an image stack in DICOM format (pixel size: 5.8 µm for the X4 objective, and 1.9 µm for the X10 objective). Three‐dimensional brain models were generated by manual segmentation of µCT image stacks using “Amira” software (FEI Visualization Science Group, Burlington, VT, USA) (Sombke et al. 2015). Brain structures, including neuropils, nerves, and cell clusters, were manually segmented for volumetric analysis and visualization, then an unconstrained smoothing was applied to surfaces (Amira: SurfaceGen). Additionally, the overall visualization of specific brain structures within the cephalon was achieved by employing the Amira Volren module.

### 2.7 Nomenclature

The nomenclature used to describe the neuroanatomical structures here is based on that of D. C. Sandeman et al. (1993) and Richter et al. (2010), with adjustments according to Loesel et al. (2013) and Krieger et al. (2015). In accordance with Richter et al. (2010), we prefer the term “brain” over “cerebrum.” Furthermore, we use the term “visual neuropils” to refer to “lamina,” “medulla,” and “lobula,” in line with Krieger et al. (2015). Yet, instead of using the term “lobula plate,” we refer to this structure as “lobula satellite neuropil” because the identity of this neuropil in crustaceans versus *hexapods* is discussed controversially (e.g., Strausfeld 2005; 2021; Strausfeld and Olea‐Rowe 2021). The traditional term “hemiellipsoid body” (Schmidt 2015) was modified to “mushroom body” to reflect the accumulating evidence suggesting that these structures are equivalent to the insect MBs (Strausfeld 2020; Strausfeld et al. 2020; Strausfeld and Sayre 2020, 2021; Harzsch and Krieger 2021). Because the segmental composition of the arthropod “syncerebrum” (sensu Richter et al. 2010) is a matter of endless dispute (reviewed in Scholtz 2015) and new evidence related to this problem is still emerging (e.g., Nel et al. 2025, Strausfeld et al. 2025), we refrain from using the terms “protocerebrum,” “deutocerebrum,” and “tritocerebrum” to designate brain units.

### 2.8 Measurements

The volumes of selected neuropils for both brain hemispheres (OLs, LANs, A2Ns, medulla, lobula, and MB/TM complex) relative to total brain volume were measured from 3D reconstructions with Amira's material statistics tool for one specimen per species (*n* = 1). The median protocerebral neuropil (MPN) is unpaired, resulting in only one value per studied brain. For the total brain volume, the brain was delimited where antennal nerves and oesophageal connectives separate from the cerebrum. In *P. elegans*, the volume of the eyestalk tissue linking the lateral protocerebrum with the central brain was omitted from the total brain volume, as this region contains no neuropil or cell clusters. Excluding this portion allows a more accurate comparison with *R. exoculata*, which does not possess eyestalks.

For the volume and number of olfactory glomeruli, measurements and estimations were made from sections from one specimen per species (*n* = 1), revealed with synapsin immunoreactivity as described in Beltz et al. (2003).

## 3 Results

### 3.1 General Anatomy of the Brain

**FIGURE 2:**
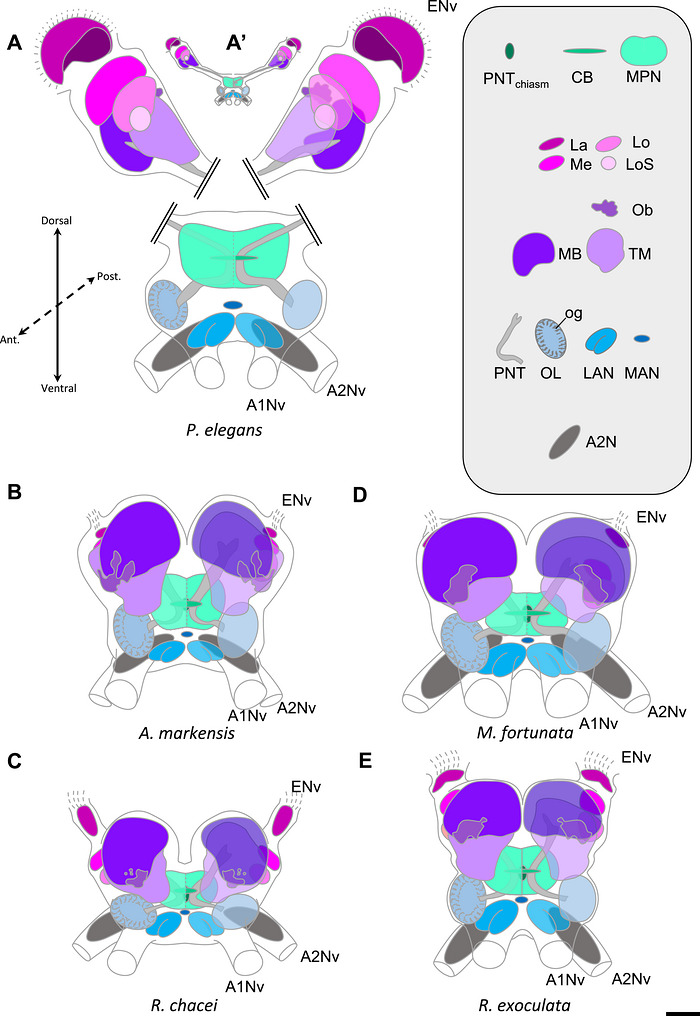
Frontal overview (with transparent right half view) of brain anatomy and major neuropils of the four alvinocaridid shrimps for the Mid‐Atlantic Ridge and of a phylogenetically close relative species of coastal Caridea. (A) *Palaemon elegans*. (A’) *Palaemon elegans* (Large overview). (B) *Alvinocaris markensis*. (C) *Mirocaris fortunata*. (D) *Rimicaris chacei*. (E) *Rimicaris exoculata*. A2N, antenna 2 neuropil; A1Nv, antenna 1 nerve; A2Nv, antenna 2 nerve; CB, central body; ENv, eye nerve; La, lamina; LAN, lateral antenna 1 neuropil; Lo, lobula; LoS, lobula satellite neuropil; MAN, median antenna 1 neuropil; MB, mushroom body; Me, medulla; MPN, median protocerebral neuropil; Ob, onion bodies; og, olfactory glomerulus; OL, olfactory lobe; PNT, projection neuron tract; TM, terminal medulla. Scale bar = 100 µm.

In the hydrothermal vent species, the brains form a single compact mass within the cephalothorax, whereas in *P. elegans*, the visual neuropils and medulla terminalis are located in the eyestalk, at some distance from the central brain (Figure 2A,A’). Unlike the other species, *R. chacei* has a more flattened arrangement of brain areas on the dorsoventral axis, with OLs inclined more horizontally (Figure 2D).

The MPN is placed at the center of the brain, right posterior to the MBs and TMs. It embraces two medially fused parts, the anteriorly located protocerebral bridge (PB), which is posteriorly followed by the central body (CB). The PNTs form a chiasm close to the CB (PNT chiasm). This tract connects the OLs to the secondary processing complex within the protocerebrum, MBs, and TMs (Figure 2A–E).

Further posteriorly, the (bilaterally paired) OLs, as well as the (bilaterally paired) LANs, are located. Another neuropil, the unpaired median antennular neuropil (MAN), is located posterior to the LANs (Figure 2A–E). These neuropils seem to display the same morphology between the different hydrothermal vent shrimps, as well as in *P. elegans* (Figure 2A–E). The (bilaterally paired) A2Ns are positioned more posteroventrally to the LANs and OLs. They do not show any obvious morphological disparities between species, even between hydrothermal vent shrimps and *P. elegans* (Figure 2A–E).

### 3.2 Visual Neuropils and Retinal Arrangement

**FIGURE 3:**
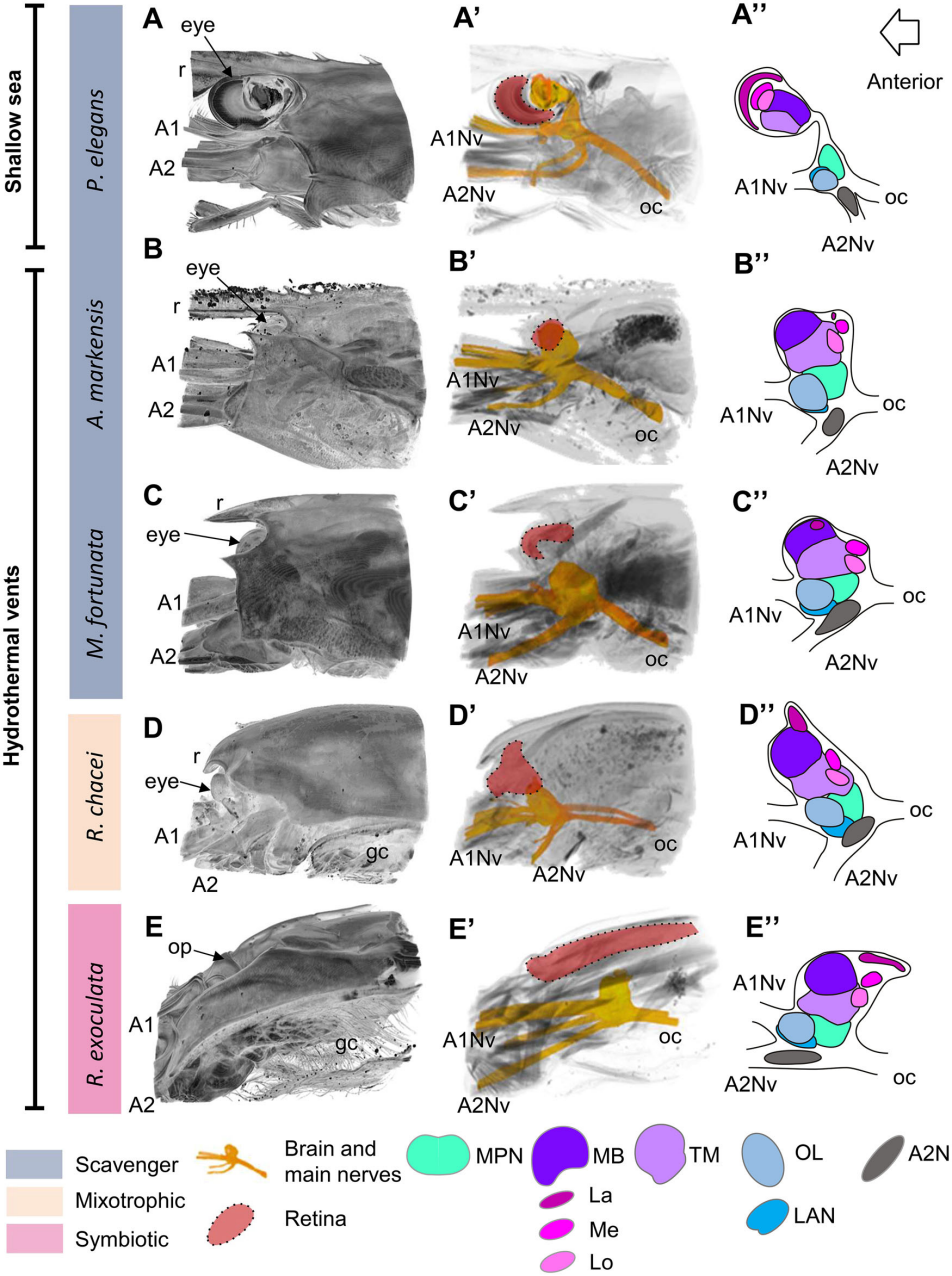
µCT scan lateral overview of cephalothorax and brain anatomy of the four alvinocaridid shrimps from the Mid‐Atlantic Ridge and of a phylogenetically close relative species of coastal Caridea. (A) *Palaemon elegans*. (B) *Alvinocaris markensis*. (C) *Mirocaris fortunata*. (D) *Rimicaris chacei*. (E) *Rimicaris exoculata*. A2N, Antenna 2 neuropil; A1Nv, antenna 1 nerve; A2Nv, antenna 2 nerve; gc, gills chamber; La, lamina; LAN, lateral antenna 1 neuropil; Lo, lobula; MB, mushroom body; Me, medulla; MPN, median protocerebral neuropil; oc, oesophageal connectives; OL, olfactory lobe; op, ocular plate; r, rostrum; TM, terminal medulla.

In hydrothermal vent shrimps, the visual neuropils comprise three successive elements, which, from proximal to distal, are the lamina (La), medulla (Me), and lobula (Lo) (Figures 2B–E and 3A″–E″). The lamina receives several axon bundles from the retina, the eye nerve (ENv; Figure 2B–E). These axon bundles are more condensed due to the size of the lamina in hydrothermal vent shrimps, compared to their coastal relative, *P. elegans* (Figure 2A–E). The medulla, more rounded in shape, is interconnected by fibers to both the lamina and the lobula. No lobula satellite neuropil was observed in all hydrothermal vent species, whereas this neuropil is present in their coastal relative, *P. elegans* (Figure 2A–E).

The retina as well as the more distal visual neuropil (i.e., lamina) appear to be more developed in *R. exoculata* and *R. chacei* than in the other hydrothermal vent species. In *R. exoculata*, the retina is elongated dorsally along the cephalothorax, while in *R. chacei*, it lines the back of the eyes and the dorsal anterior part of the cephalothorax (Figure 3D′,E′). These characteristics are also important in the coastal species *P. elegans*, with a hypertrophied lamina located behind a wide retina lining the eyes (Figure 3A′). The lamina is continuous with the retina in the two *Rimicaris* species, with a posterodorsal position in *R. exoculata* and a more anterodorsal position in *R. chacei* (Figure 3D″,E″). In the scavenger species *M. fortunata*, the lamina appears much less developed and even less in *A. markensis* (Figure 3B″,C″).

### 3.3 OB

**FIGURE 4:**
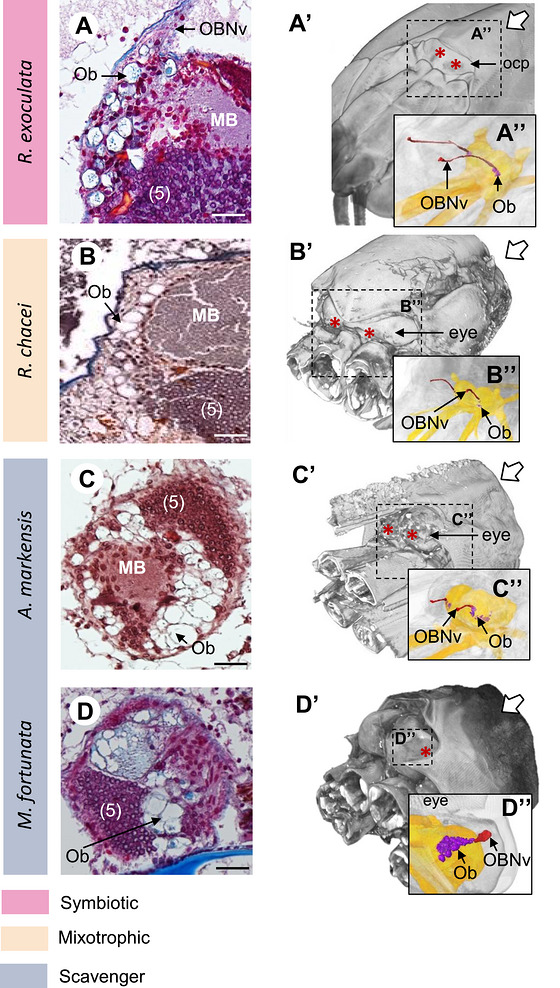
Overview of the organ of Bellonci in the four alvinocaridid shrimps of the Mid‐Atlantic Ridge. Retina paraffin section of (A) *Rimicaris chacei*, (B) *Rimicaris exoculata*, (C) *Mirocaris fortunata*, and (D) *Alvinocaris markensis*. Volume rendering of the µCT dataset showing the cephalothorax of (A′) *Rimicaris exoculata*, (B′) *Rimicaris chacei*, (C′) *Alvinocaris markensis*, and (D′) *Mirocaris fortunata*. Brain overview of (A″) *Rimicaris exoculata*, (B″) *Rimicaris chacei*, (C″) *Alvinocaris markensis*, and (D″) *Mirocaris fortunata*. MB, mushroom body; OBNv, organ of Bellonci nerve; Ob, onion bodies; ocp, ocular plate; *, Bellonci's organ nerve location on cephalothorax view; (5), globuli cell cluster. Scale bars = 100 µm. White arrows: anterior part of the cephalothorax.

In hydrothermal vent species, this organ is well developed and located close to the MB and TM, as already noted by Charmantier‐Daures and Segonzac 1998. The OB comprises numerous conspicuous “onion bodies” (Ob), the number of which we estimated to be around 50 per organ, as determined by the analysis of successive histological sections. The organ is connected to a nerve tract (OBNv), and the onion bodies are bordered by densely distributed granular cells (Figure 4A–D,A″–D″). These cell bodies are positioned anterolaterally to the MBs (Figure 4A″–D″). The nerve associated with the OB seems to originate beneath the cuticle of the eye plate in *R. exoculata*, and beneath the cuticle of the eye in the other three hydrothermal vent species, then progresses through the retina and extends further toward the brain, close to the MB (Figure 4A’–D’). This nerve also appears denser and shorter in *M. fortunata*, whereas it is thin and long in the other hydrothermal vent shrimps (Figure 4D″). Unfortunately, we did not have enough properly fixed material to study the area of origin of this nerve in any detail so that we cannot comment on the possible presence of a sensory pore organ close to the eyes (see Section 4).

### 3.4 MB and TM

**FIGURE 5:**
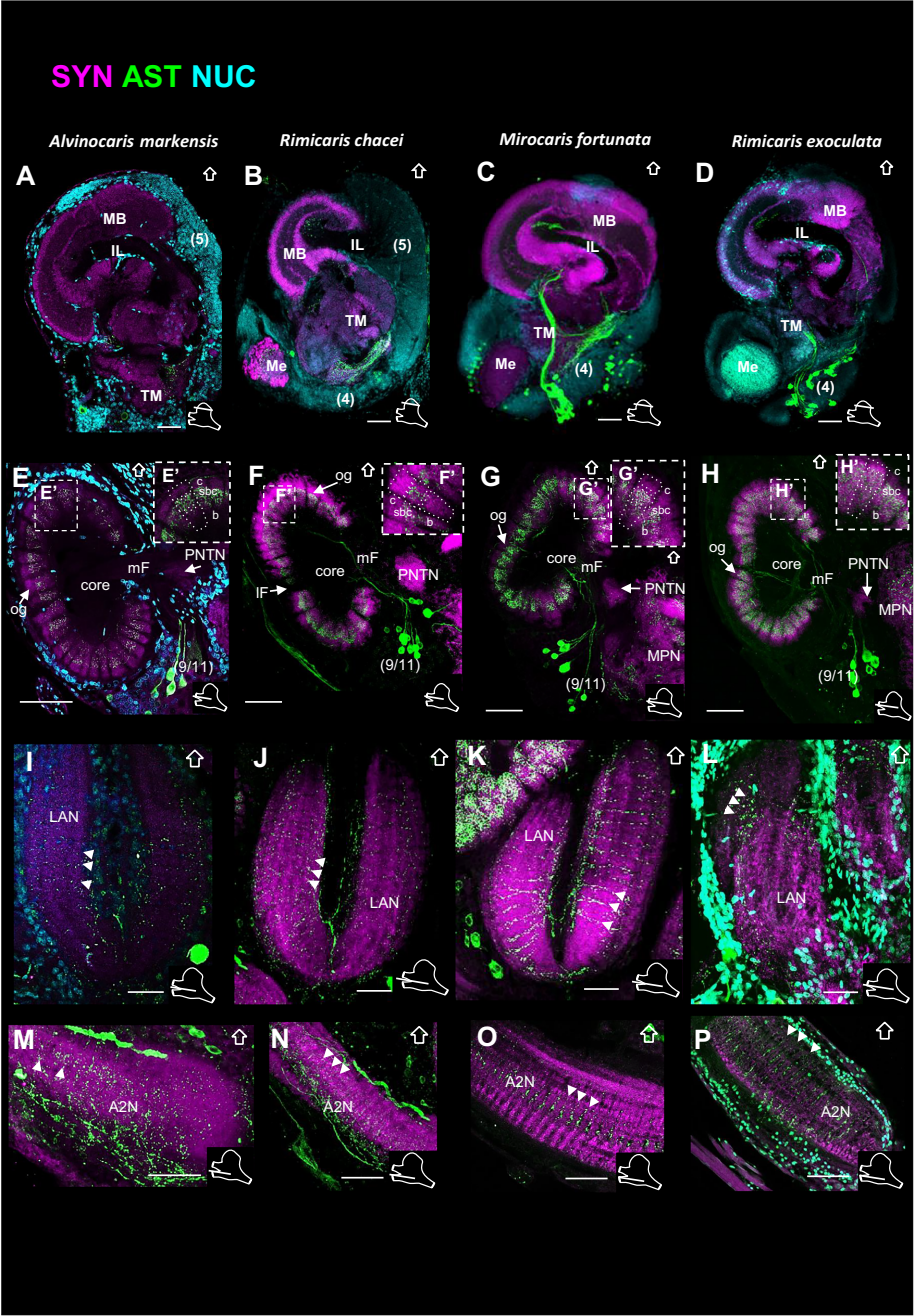
Micrographs of horizontal brain sections of the mushroom body and terminal medulla, olfactory lobes, lateral antenna 1 neuropil, and antenna 2 neuropil, from dorsal to ventral, triple‐labeled for synapsin immunoreactivity (SYN, magenta), allatostatin‐like immunoreactivity (AST, green), and a nuclear marker (NUC, cyan), in four alvinocaridid shrimps: *Alvinocaris markensis* (A, E, I, M), *Mirocaris fortunata* (C, G, K, O), *Rimicaris chacei* (B, F, J, N), and *Rimicaris exoculata* (D, H, L, P). (A–D) Lateral protocerebrum. (E–H) Olfactory lobe. (E’–H’) Focus on the olfactory glomerulus. (I–L) Lateral antennular neuropil. (M–P) Antenna 2 neuropils. (4), cell clusters; (9/11), olfactory interneurons; A2N, antenna 2 neuropil; ASTir, allatostatin‐like immunoreactivity; b, base region of the olfactory glomerulus; c, cap region of the olfactory glomerulus; IL, intermediate layer; LAN, lateral antenna 1 neuropil; lF, lateral foramen; MB, mushroom body; Me, medulla; mF, median foramen; MPN, median protocerebral neuropil; og, olfactory glomerulus; OL, olfactory lobe; PNTN, projection neuron tract neuropil; sbc, subcap region of the olfactory glomerulus; SYNir, synapsin immunoreactivity; TM, terminal medulla neuropil. Scale bars = 100 µm. White arrowheads: striation of neuropil (LAN or A2N) visible by allatostatin immunoreactivity. White arrows: anterior part of the brain.

The MB and TM, highly developed in the hydrothermal vent shrimps, share the same structure in the four alvinocaridid species. The MB is divided into two parts: a hemispherical cupola and a torus fused posteriorly to the TM. These two regions are separated by an intermediate layer (IL) receiving afferent fibers anteriorly from the TM (Figure 5A–D) and neurites from the cell cluster (4) (Figure 5A–D), displaying allatostatin‐like immunoreactivity (ASTir) near the cap region (Figure 5C). On the anterior side, this second region is slightly spherical in shape and is attached to the MB (Figures 2 and 5A–D). Its posterior part is defined in these species by a large network of unstructured fibers (Figures 2 and 5A–D). This complex receives input from the OLs via the PNT in the four species (Figure 2B–E).

### 3.5 OLs and LANs

In *R. exoculata*, the (bilaterally paired) OLs consist of radially arranged conical olfactory glomeruli around a central, synapse‐free core (Figure 5A–H). These olfactory glomeruli show SYNir and ASTir, defining cap, subcap, and base regions (Figure 5E–H). Strong ASTir labeling is observed in local olfactory interneurons (cell cluster 9), numbering ∼50 per side, with soma diameters of 15–20 µm. Their neurites project through the median foramen into the glomeruli, especially into the subcap region, where fine ASTir‐positive processes are prominent. Rather than converging at the base, these fibers enter at specific points and spread laterally within the subcap. A second group of ASTir neurons (cluster 11), with soma diameters around 20 µm, surrounds the OLs without projecting into them. Other clusters forming the olfactory globular tract lack ASTir but are partially visible as they enter the projection tract neuropil (PNTN) (e.g., Figure 5H). Across species, OL organization varies in terms of numbers of olfactory glomeruli: *A. markensis* has slightly more glomeruli, *R. exoculata* has the fewest and the smallest mean glomerular volume, and *R. chacei* shows the highest mean glomerular volume but smaller OL volume overall (Table 2).

**TABLE 2:** Summary table of mean measurements for aesthetasc characteristics, olfactory lobe characteristics, and total brain volumes from hydrothermal vent species and coastal relative *Palaemon elegans*. Aesthetasc number and dimensions are from Zbinden et al. (2017). The volumes of the neuropils are obtained from µCT reconstructions performed with Amira software. For the mean volume and number of the olfactory glomeruli, measurements and estimations were made from sections revealed by synapsin immunoreactivity as described in Beltz et al. (2003). All data were measured for one individual per species.

	Aesthetascs		Olfactory lobes			Total brain volume (mm^3^)
Species (body length)	Total number	Length × diameter (µm)	Neuropil total volume (× 10^6^ µm^3^)	Mean Glomerular volume (× 10^3^ µm^3^)	Glomeruli number (L+R OL)	
*Rimicaris exoculata* (5.5 cm)	206	20 × 170	150	153	372	1.179
*Rimicaris chacei* (5.5 cm)	226	19 × 251	104	272	466	0.562
*Mirocaris fortunata* (3 cm)	120	16 × 234	280	245	399	1.308
*Alvinocaris markensis* (8.2 cm)	220	21 × 531	185	176	650	0.892
*Palaemon elegans* (7 cm)	280	14 × 230	190	225	531	0.927

The (bilaterally paired) LANs do not appear to vary morphologically among species and have the same general shape with a medial split (Figures 2B–E and 5I–L). ASTir neurons can be observed anteromedially to the LANs in the form of thin neurites extending between the OLs and LANs (Figure 5I–L). The latter exhibited a faintly striated distribution of ASTir material, with more distinct neurites extending anteroposteriorly along the neuropil (Figure 5I–L). Transverse networks of neurites showing ASTir seem to originate from the neurite extending anteroposteriorly along the LAN (Figure 5I–L).

### 3.6 A2N

**FIGURE 6:**
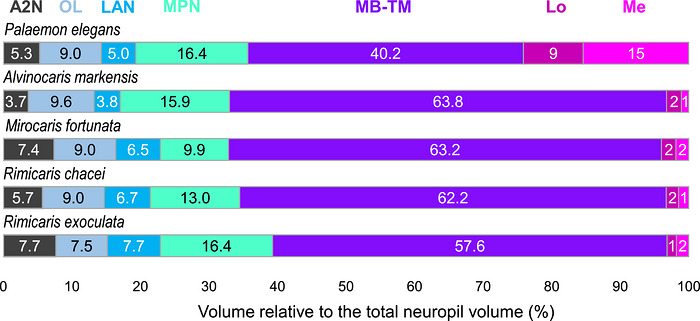
Relative volumes of paired neuropils compared to total neuropil volumes (in percentage). A2N, antenna 2 neuropil; LAN, lateral antenna 1 neuropil; Lo, lobula; Me, medulla; MB, mushroom body; MPN, median protocerebral neuropil; OL, olfactory lobe; TM, terminal medulla neuropil.

The (bilaterally paired) A2Ns show the same cylindrical shape between hydrothermal vent species (Figures 2B–E and 5M–P), as well as in *P. elegans* (Figure 2A). A transverse striated organization similar to that observed in LANs is also present in the different species, with associated networks of neurites showing ASTir. As for LANs, distinct neurites seem to extend anteroposteriorly along the neuropil and innervate the transversal striation with smaller ASTir material (Figure 5M–P). The proportion of these two neuropils (LAN and A2N) within the total neuropil volume differs. While *A. markensis* has the smallest LANs and A2Ns (3.7%–3.8% of the total neuropil volume each), *R. exoculata* has the largest ones (7.7% each; Figure 6). The other species fall in between these extremes.

### 3.7 Neuropil Volumes

The total brain volume shows marked differences between species. *Rimicaris exoculata* has a brain twice as large as that of *R. chacei*, even though they are the same size. *Mirocaris fortunata*, the smallest of the four shrimp species, has the largest brain. Concerning *P. elegans* and *A. markensis*, data show similar brain volumes, which are around 0.9 mm^3^ (Table 2).

The relative volumes of the neuropils (i.e., compared to total neuropil volume) in *R. chacei*, *M. fortunata*, and *A. markensis* are similar to those in *R. exoculata*. In the MAR hydrothermal vent species, the total volume of visual neuropils does not exceed 4% of the total brain volume, compared with 24% in the coastal species *P. elegans* (Figure 6). Compared to the visual neuropils, the complex of MB and TM occupies more than half of the brain, making up about 57%–63% of the total neuropil volume. In contrast, it accounts for only about 40% in the coastal species *P. elegans* (Figure 6). The more posterior neuropils (OLs, LANs, A2Ns) together represent about 20% of brain volume. Their size shows little variation among the four hydrothermal vent species and is comparable to that of *P. elegans* (Figure 6). Among the hydrothermal vent shrimps, *A. markensis* has the smallest neuropils associated with the antennula (antenna 1) and antenna (antenna 2), with the A2N making up 3.7% and the LAN 3.8% of the total neuropil volume (Figure 6). *Rimicaris exoculata* shows the lowest proportion of the OLs, at 7.5% of the brain volume, where most species have a relative volume of about 9% (Figure 6). The MPN also varies in size between species. It is largest in *R. exoculata* and *P. elegans* (about 16.4%), but smaller in *R. chacei* (13%) and *M. fortunata* (9.9%) (Figure 6).

### 3.8 Myoarterial Formation

**FIGURE 7:**
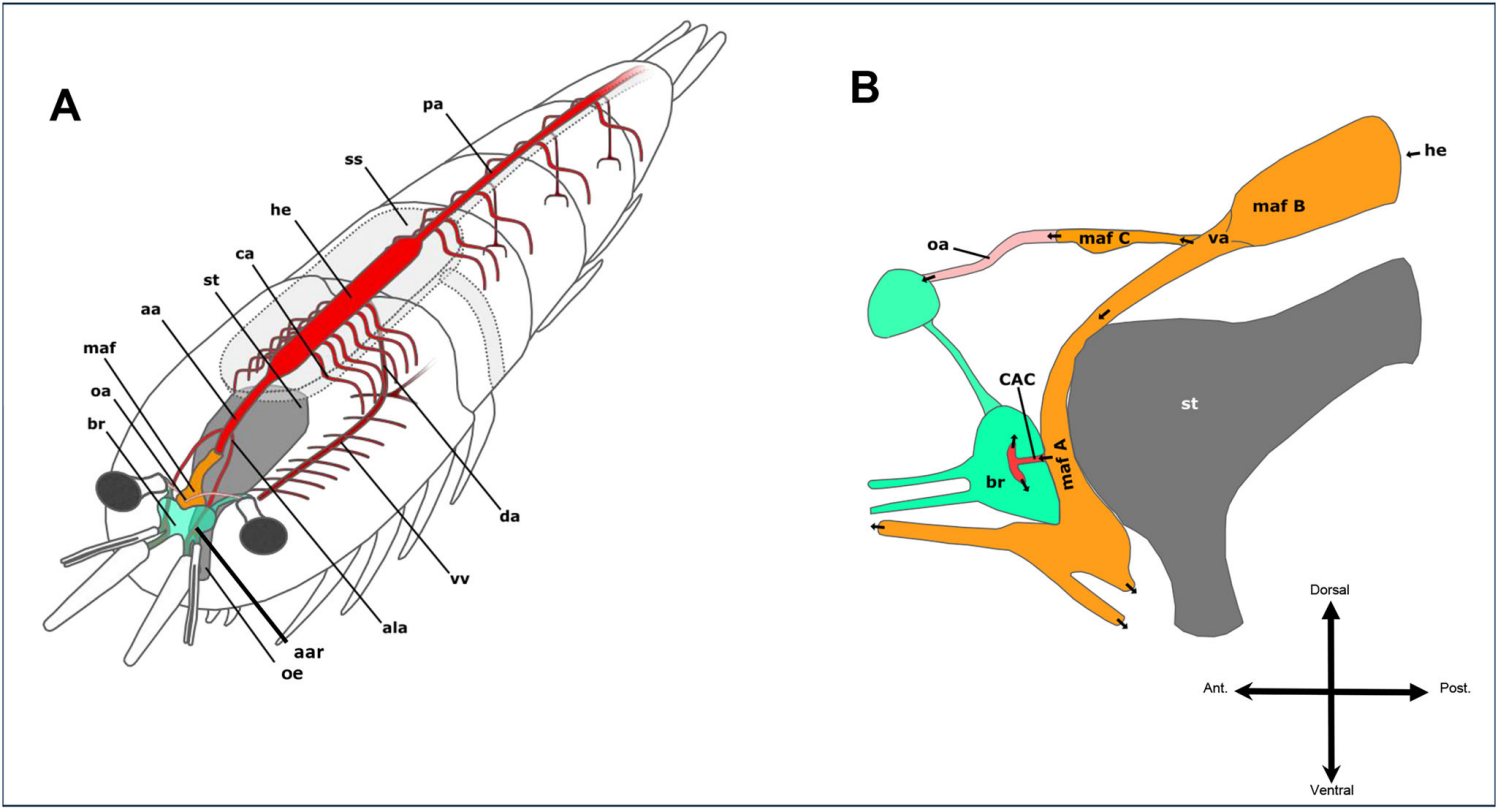
Schematic representation of the circulatory system (A) and lateral view of the myoarterial formation (B) in decapods. aa, anterior aorta; aar, antennal arteries; ala, anterior lateral arteries; br, brain; ca, cardiac arteries; CAC, central cerebral artery; da, descending artery; he, heart; maf, myoarterial formation; maf A–C, myoarterial formation A to C; oa, ophthalmic arteries; oe, esophagus; pa, posterior aorta; ss, suspending strand; st, stomach; va, valve; vv, ventral vessel. Modified from Watling et al. (2013).

**FIGURE 8:**
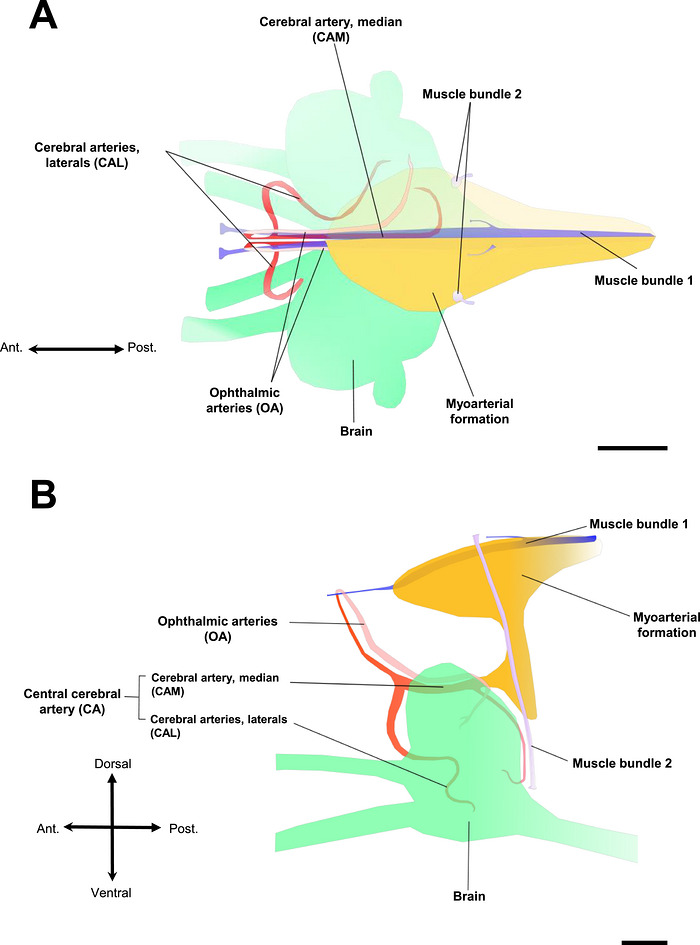
Schematic representation of the brain and myoarterial formation of MAR hydrothermal vent shrimps, followed by the arterial network providing oxygenated blood to specific areas of the brain. (A) Dorsal view. (B) Lateral view. Scale bar = 100 µm.

**FIGURE 9:**
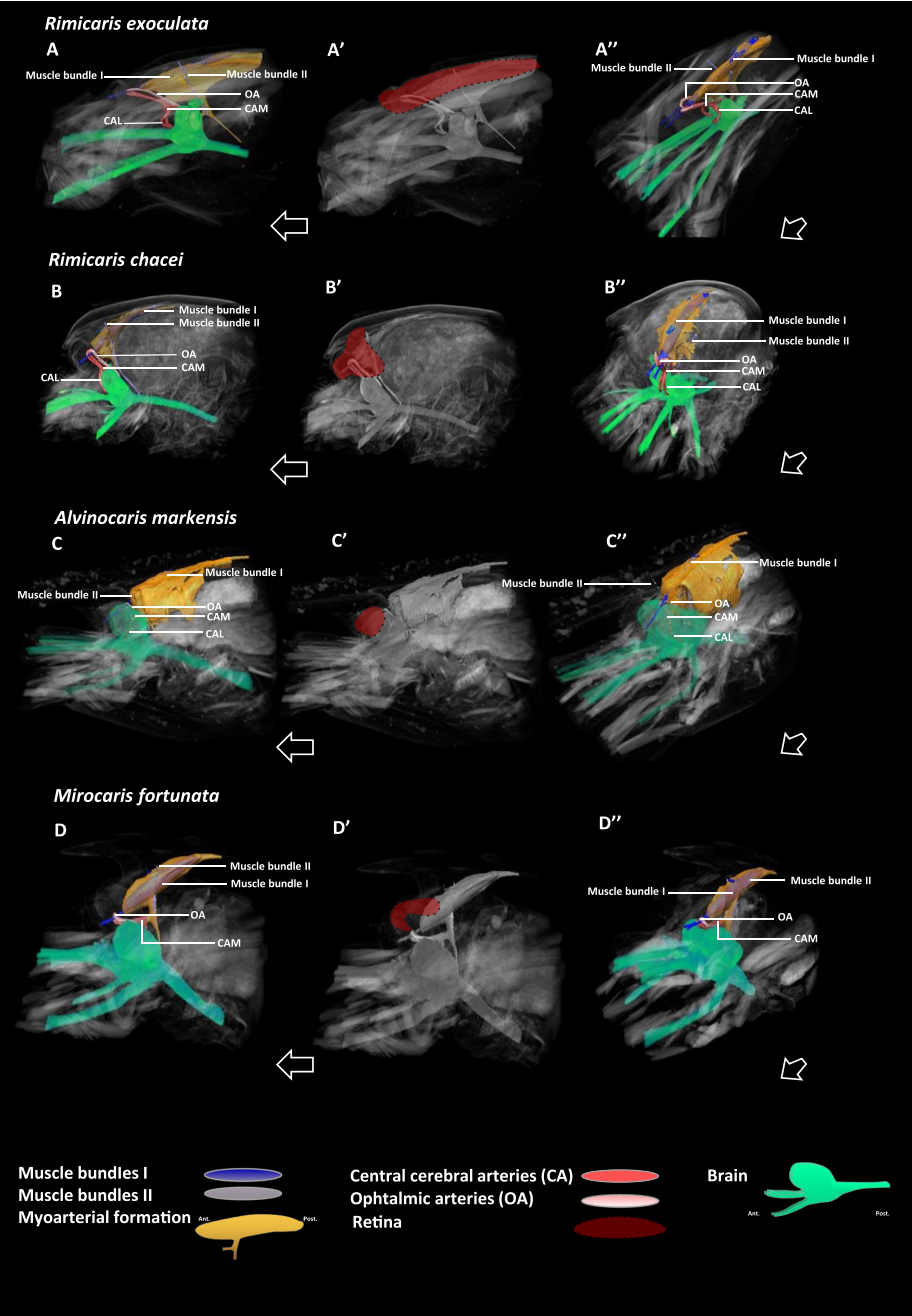
Volume rendering of the µCT dataset showing an overview of the myoarterial formation, associated muscle bundles, and arterial branches in our four alvinocaridid shrimps (A–D″). Dark blue, muscle bundle 1; dark red, retina location; light blue, muscle bundle 2; green, brain; orange, myoarterial formation; pink, ophthalmic arteries; red, central cerebral arteries. CAL, central cerebral arteries laterals; CAM, central cerebral artery, median; OA, ophthalmic arteries. (A, A′, A″) *Rimicaris exoculata*. (B, B′, B″) *Rimicaris chacei*. (C, C′, C″) *Alvinocaris markensis*. (D, D′, D″) *Mirocaris fortunata*. White arrows: anterior part of the cephalothorax.

In the previously studied decapods with stalked eyes, the *cor frontal* system targets directly specific areas of the brain with two types of arteries: (1) the OA, which are paired and supposedly targeting the protocerebrum in each eyestalk (Figure 7A,B) (Watling et al. 2013), and (2) the unpaired central cerebral artery (CA), connecting the anterior part of the myoarterial formation to the rest of the brain (Watling et al. 2013) (Figure 7B). In alvinocaridids, this formation extends along the dorsal cuticle on the anterior part of the cephalothorax, above the brain in *R. exoculata*, *R. chacei*, *A. markensis*, and *M. fortunata* (Figures 8A,B and 9A–D,A″–D″). Within this myoarterial formation, we can see the presence of two muscle bundles (mafm) (Figure 8A,B). The muscle bundle 1 extends dorsally toward the anterior part of the cephalothorax, through the myoarterial formation. These muscles are supposedly attached dorsally to the cuticle on the median part of the myoarterial formation, close to the eyes on the anterior cephalothorax, and probably posteriorly at the end of the myoarterial formation (Figures 8A,B and 9A–D,A″–D″). However, µCT scan data do not allow for accurate identification of the exact attachment points to the cuticle. Other, finer muscle bundles (i.e., muscle bundle 2) (Figure 8A,B) cross the myoarterial formation on its median part (perpendicularly) and are supposedly attached to the dorsal part of the cuticle of the cephalothorax, as for muscle bundle 1 (Figures 8A,B and 9A–D,A″–D″). However, µCT scans of the samples do not allow proper observation of the rest of this muscle bundle 2, but we suppose that this structure extends and attaches ventrally to the cuticle of the cephalothorax beneath the brain, as observed in some species such as *R. chacei*, with a muscle bundle 2 appearing to extend under the brain (Figure 9B).

The size of this organ varies according to the species observed. In *R. exoculata*, it is as long as the retina and extends ventrally, overlapping the dorsal region of the brain (Figure 9A,A″). This position is similar in *R. chacei* and *M. fortunata*. In *A. markensis*, the MAF seems to be larger and spreads ventrolaterally behind the protocerebrum (Figure 9C).

**FIGURE 10:**
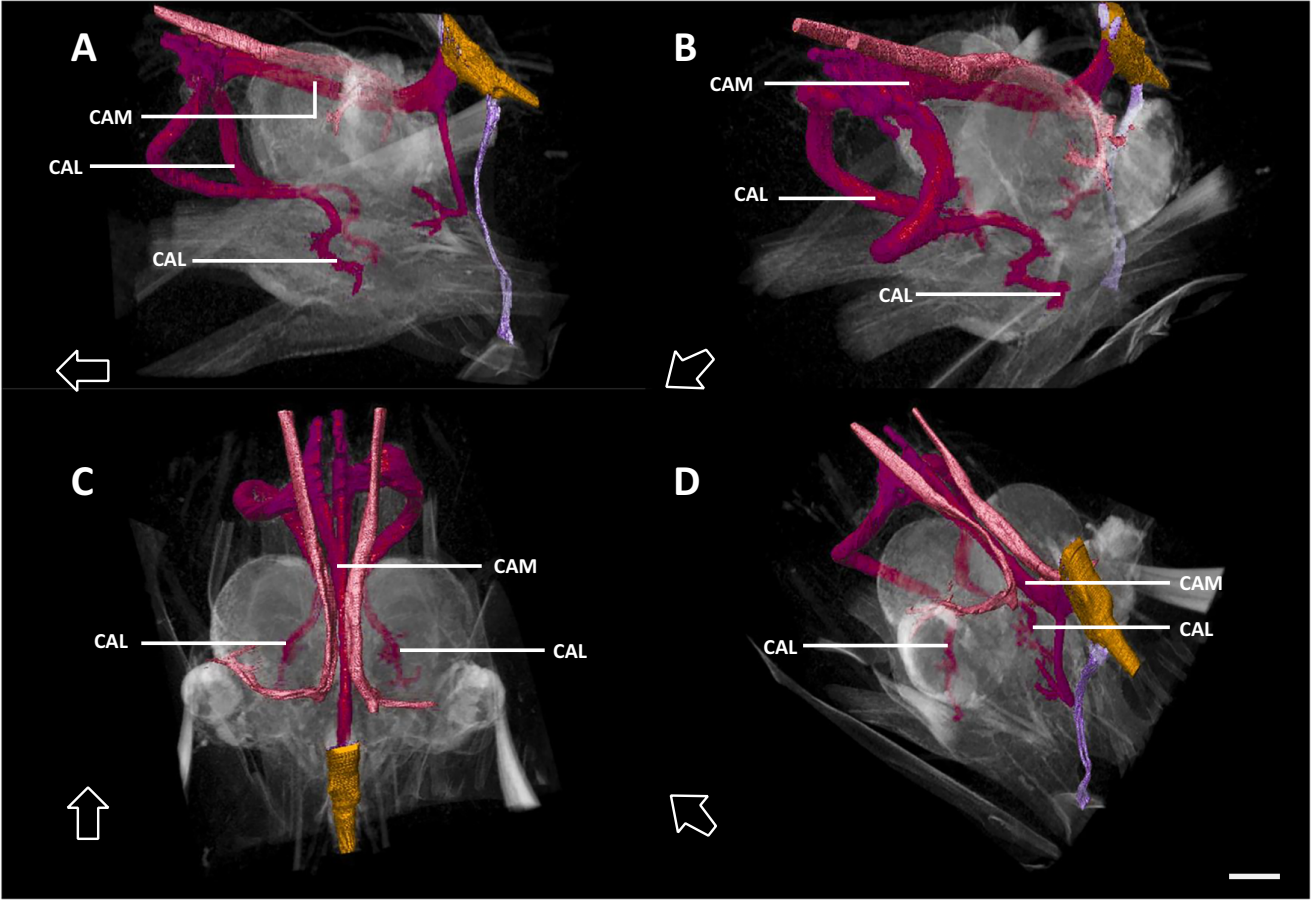
Volume rendering of the µCT dataset showing the detailed development of the arterial branches of *Rimicaris exoculata* around the central nervous system. Dark red, central cerebral arteries (CA); gray, brain; orange, myoarterial formation (MaF); pink, ophthalmic arteries (OA); purple, muscle bundles 2; CAL, central artery, lateral; CAM, central artery, median. Scale bar = 100 µm. White arrows: anterior part of the cephalothorax.

In *R. exoculata*, the myoarterial formation subdivides anteriorly near the eyes to give rise to three arteries extending dorsally. One central CA divided into three smaller arteries: a median (CAM) passing over the brain between the two MBs, then dividing into two branches; another one penetrating behind the brain toward the MPN, while the larger part of the CAM is merging with the ventral region of the myoarterial formation; furthermore, two lateral branches (CAL) extending toward the OLs and LANs (Figures 2E, 8A,B, 9, and 10A–D). The central CA also extends around the two muscle bundles (i.e., muscle bundle 1), extending longitudinally through the myoarterial formation, subsequently dividing into two OAs. These OAs make a half‐turn at the back of the brain and connect posterodorsally with the visual neuropils and the MPN (Figures 2E, 8A,B, 9A, and 10A–D).

In *R. chacei* and *R. exoculata*, the central CA appears to subdivide before making a U‐turn around the muscle bundle 1 to form the OAs, whereas in the other two species, this artery appears to have a compact structure over much of its length (Figures 9A–D and 10C). In *M. fortunata*, the subsections of the central CA that irrigate the brain (CAM and CAL) are difficult to identify in µCT scans, and the diameter of the muscle bundle 1 running longitudinally through the myoarterial formation appears to be larger (Figures 9D and 10). In *A. markensis*, this arterial system is located posteriorly to the protocerebrum, with thinner arteries than in the three other species, making them more difficult to observe (Figure 9C,C″).

## 4 Discussion

### 4.1 The Alvinocaridid Visual System

In *R. exoculata*, the architecture of the visual system has been well documented and is dominated by a pair of large eyes fused at the front of the cephalothorax, the ocular plate (Van Dover et al. 1989; O'Neil et al. 1995; Nuckley et al. 1996; Gaten et al. 1998a, 1998b). This ocular plate is located beneath the dorsal cuticle of the cephalothorax. Its broad retina is composed of an enlarged rhabdom that covers the tapetum, a reflective cell layer that enhances light absorption (Van Dover et al. 1989; Machon et al. 2019; Chamberlain 2000). In the brain, three visual neuropils, the lamina, medulla, and lobula, are located dorsally, with the lamina connected to the retina by the eye nerve. The visual neuropil's volume is smaller than in the coastal species *P. elegans* (Machon et al. 2019). The evolutionary conservation of visual neuropils contrasts with their complete absence in cave species that have adapted to a life in partial or constant darkness (e.g., Fanenbruck et al. 2004; Stegner et al. 2015). In these species, the reduction of these nervous tissues was favored by selection pressures linked to environmental light conditions (partial or constant darkness). Indeed, the elimination of unsolicited neuronal structures generates energy savings for such organisms (Moran et al. 2015). Nevertheless, the conservation of these structures in *R. exoculata* indicates a functional visual system, likely enabling the detection of faint thermal radiation near hydrothermal vents (Gaten et al. 1998a, 1998b; Chamberlain 2000).

Both *R. exoculata* and *R. chacei* have a well‐developed retina and a lamina that extends directly adjacent to it in parallel (Lakin et al. 1997). This retina is even more developed in *R. exoculata*. These modifications may help these species to perceive low‐intensity light emissions like infrared (>2500 nm) and near‐infrared (∼700–2500 nm) emitted by the vent fluids (O'Neill et al. 1995; Nuckley et al. 1996; Chamberlain 2000). Additional comparative data of the ultrastructure of the retina and wavelength spectra would be needed to confirm these claims. We have also observed complete, albeit smaller retinas with a rhabdom, a tapetum, and numerous pigment cells in *M. fortunata*, as well as in *A. markensis*. The presence of these visual structures, as well as the three visual neuropils, in these scavenger species that are not directly dependent on reduced fluid compounds for feeding, suggests different evolutionary pressures than diet for the conservation of these anatomical features in low‐light environments, such as detection of warm temperature emissions to navigate safely around vents (Lagerspetz and Vainio 2006, Ravaux et al. 2021). In other animals, heat detection is provided by a specific receptor family, the transient receptor potential (TRP) family, and particularly the TRP A subfamily (Venkatachalam and Montell 2007; Kozma et al. 2018; Himmel and Cox 2020). These TRPs were also found in the visual system of the fruit fly *D. melanogaster*, where genes from the TRPL and TRPγ subfamilies are expressed in the rhabdomere and could play a role as a light‐sensitive channel complex allowing phototransduction in the dark (Montell 2003; Cronin et al. 2006; Frolov 2016). Similar observations were made in the cockroach *Periplaneta americana* (French et al. 2015). Searching for such TRP subfamilies in the alvinocaridid visual system would be worthwhile, particularly in the genus *Rimicaris*, whose representatives show a well‐modified visual apparatus. Yet, the axon bundles (ENv) connecting the retina to the lamina appear to be more condensed in the four species of MAR hydrothermal vent shrimps than in *P. elegans*, regardless of retinal enlargement. Such an arrangement could be explained by a smaller lamina, but also by the shift of the visual neuropils to a posterodorsal position in the vent shrimps. Such an arrangement corresponds to the modification of the visual system and the link between retina and lamina while avoiding the myoarterial formation on the dorsal part of the brain, which is generally just behind the retina. Indeed, the widespread disposition of axon bundles between the retina and the lamina observed in *P. elegans* would be more difficult to maintain for species with such important structures as the myoarterial formation observed in alvinocaridids.

We also found that the lobula satellite neuropil, another visual neuropil present in several malacostracan taxa (Krieger et al. 2012; Kenning et al. 2013; Kenning and Harzsch 2013; Bengochea et al. 2017), seems to be absent in all four hydrothermal vent species studied. The absence of the lobula satellite neuropil has been suggested as a loss of capability to mediate motion detection (Sztarker et al. 2005; Machon et al. 2019). This absence in all species studied here would suggest a more general loss of ability to detect motions in alvinocaridids.

### 4.2 A Sensory Function of the OB?

The functional morphology of the OB was described in many adult malacostracan crustaceans, but its function remains enigmatic. Typical elements of the adult malacostracan OB are the onion bodies (named after the concentrical appearance of membranous lamellae) that are extensive ciliated processes linked with sensory cell bodies (Chaigneau 1973; G. Smith 1974). The onion bodies were reported to display a high level of structural plasticity depending on season, daytime versus night, and molt cycle (Bellon‐Humbert et al. 1981; Bellon‐Humbert and van Herp 1988), perhaps mirroring as yet not systematically explored differences in the physiological states of the animals examined. Photoreceptive, chemoreceptive, or baroreceptive functions have been proposed for the adult OB, as well as a mainly secretory function (reviewed in Chaigneau and Besse 1994). However, as shown by Hallberg and Kauri (1992) in *Macrobrachium rosenbergii*, the organ most likely does not have a photoreceptive function, a hypothesis that was already challenged by Chaigneau and Chataigner (1977) based on the observation that the OB is also present in crustaceans living in caves and that is in line with the observation of distinct OBs in crustaceans that live in the deep sea (Charmantier‐Daures and Segonzac 1998, and our own data). Although crustaceans typically expose their olfactory sensilla to the outside of the body to improve contact with the stimulus (Derby 2021), a potential chemosensory function of the OB cannot be ruled out. Moreover, Chaigneau (1994) suggested considering a function for measuring osmotic pressure. The OB is frequently associated with a complex of additional presumably sensory organs, the sensory pore organs (Chaigneau 1973). This close association of the two types of organs is a commonly reported theme in adult crustaceans (Kauri and Lake 1972; Chaigneau 1994; Charmantier‐Daures and Segonzac 1998), suggesting that they may cooperate in detecting several sensory modalities (reviewed in Chaigneau 1994). The alvinocaridid hydrothermal vent shrimps *R. exoculata* and *C. chacei*, and the crab *Segonzacia mesatlantica* possess conspicuous OBs with distinct onion bodies, located close to the complex of TM and MB (Charmantier‐Daures and Segonzac 1998). About 50 onion bodies were suggested to be present on each side of the brain of *R. exoculata* (Machon et al. 2019), and we estimate that similar numbers may characterize the other shrimp species studied here. A nervous connection of the OB with a cuticular pore was found in *R. exoculata* and *R. chacei* (Charmantier‐Daures and Segonzac 1998; Machon et al. 2019). If in alvinocaridids we face a multifunctional sensory organ, a chemosensory function may be expected, perhaps to detect essential molecules such as oxygen or sulfides, and, additionally, baroreception may be another possible function of this organ complex. These hypotheses remain to be experimentally tested.

### 4.3 MB, TM, and Complex Behaviors

In malacostracan crustaceans, the complex of the TM and MB receives input from both visual and olfactory pathways and other sensory systems. These structures contain interneurons responding to several different sensory modalities and seem to function as higher integrative centers (for review, see D. C. Sandeman et al. 2014). Neuroanatomical evidence strongly suggests a close morphological and possible functional correspondence of hexapod and crustacean MBs (Strausfeld 2020; Strausfeld and Sayre 2020, 2021; Strausfeld et al. 2020). Acting as higher‐order olfactory neuropils, one of the hexapod MBs’ primary role is in odor discrimination (e.g., Galizia 2014; Farris and van Dycke 2015; Sachse and Hansson 2016), but in hexapods, these structures also receive highly processed gustatory, acoustic, visual, and mechanosensory input and are known to function in processing multimodal sensory cues in the context of odor representation (Farris 2005, 2011; Heuer et al. 2012; Strausfeld 2012; Szyszka and Galizia 2015). They also play a role in olfactory memory formation and consolidation, multimodal learning, and place memory (Fahrbach 2006; Farris 2011; Martin et al. 2011; Menzel 2014; Stopfer 2014; Menzel and Greggers 2015; Wolff and Strausfeld 2016). Likewise, in malacostracan crustaceans, these neuropils’ functions may involve more sophisticated processing related to orientation within the environment during homing or migration, recognition of suitable mating partners, and social interactions (reviewed in D. C. Sandeman et al. 2014; Harzsch and Krieger 2021). Comparative neuroanatomical studies on malacostracans have discussed a possible involvement of MBs in place memory (Krieger et al. 2010; Wolff et al. 2017; Machon et al. 2019; Strausfeld et al. 2020). In a brachyuran crab, higher integrative neuropils closely associated with, but not identical to, the MBs (Thoen et al. 2020) are also involved in memory processes (Maza et al. 2016, Maza et al. 2021). Compared to other malacostracan crustaceans, all four species of vent shrimps examined here showed a hypertrophy of both the MB and TM, with both neuropils together accounting for around 50%–60% of total brain volume in the vent species (compare Machon et al. 2019), but only around 40% in the coastal *P. elegans*. The large volume of these two neuropils in the alvinocaridid species underscores the importance of this brain region for the behavior of hydrothermal vent shrimps. We speculate that the hypertrophy of these neuropils indicates an enhanced ability to remember the location of specific sensory stimuli, such as temperature and chemical cues, within their immediate environment. In contrast to alvinocaridids, *P. elegans* shows a less developed MB but relatively larger visual acquisition neuropils, suggesting divergent sensory strategies between coastal and hydrothermal vent species. This difference likely reflects the different availability and robustness of sensory signals in hydrothermal environments compared to visually rich coastal habitats.

### 4.4 Olfactory Performance

We did not observe any consistent size effect on glomerular volumes and OL volumes of the four species studied. However, the smaller glomerular volumes and lower number of olfactory glomeruli in *R. exoculata* suggest a limited sensitivity of the system, and a lower odor discrimination ability than the other studied species. Compared to *R. exoculata*, the three other species, by their strictly or partially scavenger lifestyle, may need to detect odors as diverse as their potential food sources (e.g., carrions) in their immediate environment. However, we do not yet know the exact factors influencing the number of the olfactory glomeruli in decapod crustaceans (discussed in Harzsch and Krieger 2018).

### 4.5 Neurovascular System and Oxygen Delivery to the Brain

One of the main morphological differences observed among the four hydrothermal vent species is localized around the brain. The myoarterial formation (*cor frontale*) (for review, see Steinacker 1978; Wirkner and Richter 2013; Davie et al. 2015) is a dilatation of the anterior aorta that pumps the hemolymph specifically toward the cephalic nervous system, thereby providing it with oxygenated hemolymph (McGaw 2005; Machon et al. 2019; Watling et al. 2013). In *R. exoculata*, the myoarterial formation and its arteries differ from those described in other malacostracans because of the loss of eyestalks (McGaw 2005; Scholz et al. 2018; Machon et al. 2019). This absence of eyestalks and the compacting of brain areas seem to have modified the organization of the different cerebral arteries in that the central cerebral arteries and the OAs fused in a loop system around the muscle bundle 1 linked to the myoarterial formation. The half‐turn at the back of the brain of the OA around the muscle bundle 1 allows the development of secondary arterial networks (i.e., CAL, CAM) in different areas of the brain in hydrothermal vent species. Yet, in other decapods, specific areas like the visual neuropils, the MB, and the TM within the eyestalks are directly supplied with arteries emerging independently from the myoarterial formation, like the OA or the central artery for the back of the brain. This reorganization of the circulatory system could improve the delivery of oxygen to the brain in alvinocaridids by the hypertrophy of the muscle bundle 1, which generates greater force to pump hemolymph to the brain (Watling et al. 2013). This could favor a stable rate of oxygenated hemolymph supply from the heart, even with increasing temperatures that reduce the amount of dissolved oxygen in water (Eheart and Park 1989). Associated with the high affinity of hemocyanin to oxygen in vent shrimps (Lallier and Truchot 1997) and its insensitivity to temperature variations, this system could possibly have an advantage in providing higher rates of oxygen to the brain to compensate for hypoxia in the environment. However, this statement needs to be better evaluated. Even if these species evolve in environments where the mixing of anoxic vent fluids with the ambient seawater can create local hypoxic conditions, no data have reported anoxic conditions nor physiological hypoxia in alvinocaridids, and more specifically in *R. exoculata* swarms, which live closer to the vent fluids than the other three species (Zbinden et al. 2004). Nonetheless, Zbinden et al. (2004) describe a lower oxygen concentration (63–236 µM) than the surrounding seawater (242 µM) in water samples collected in *R. exoculata* swarms (Rainbow vent site 36°14.0′N, MAR, 2300 m depth). This may explain possible intermediate conditions of oxygen concentration in swarms compared to vent fluids (12.4 µM) and the surrounding seawater (242 µM), therefore requiring the organism to demand higher amounts of oxygenated hemolymph to survive under conditions of lower oxygen concentration. Then, the closer these species get to the fluids, the more likely they are to encounter occasional events of hypoxia.

Another important point, the concentration of oxygen in MAR hydrothermal sites (i.e., Logatchev vent site, 14°45′N, MAR, 2910–3060 m depth; Rainbow vent site 36°14.0′N, MAR, 2300 m depth) ranges from around 7.14 to 7.68 mg/L, which falls within the normoxic range typically observed in the ocean, that is, 7–8 mg/L (Ravaux et al. 2003; Zielinski et al. 2011). These results suggest that episodes of hypoxia would be more likely to occur near the vent fluid where swarms of *R. exoculata* are present, as previously suggested. However, dissolved oxygen concentrations can vary depending on numerous factors (e.g., biological activity, hydrodynamics, pollution; Eheart and Park 1989). We cannot, therefore, rule out the possibility of localized and sporadic episodes of hypoxia caused by factors like currents, seismic activity, or turbidity.

Although *R. chacei* has a less well‐developed myoarterial formation along the dorsoventral axis than *R. exoculata*, it has a more developed muscle bundle 1 that could facilitate hemolymph delivery to the brain by generating a greater hemolymph flow. These variations may reflect different adaptive strategies for the same environmental constraints. Indeed, the similarities in the arrangement of the arteries between the two *Rimicaris* species may be indicative of a similar requirement for higher oxygen supply to cope with a potential local hypoxia in their immediate environment. *Mirocaris fortunata*, although its myoarterial formation is less extensive than that of *A. markensis*, has a more developed muscle bundle 1. This feature could also influence the supply of hemolymph to the brain, as has been postulated by Machon et al. (2019) in the case of *R. exoculata*. This species is the smallest of the four biological models; in smaller animals, the metabolic rates are more important, and therefore, a greater amount of energy is required. The development of this muscle bundle 1 may be a response to higher energy requirements caused by the metabolism, even though living conditions are similar to those of *A. markensis*.

*Alvinocaris markensis* demonstrated an enlargement of the myoarterial formation, a reduction in the size of the associated arterial branches, as well as an invagination of this network into the protocerebrum. In the other three species, however, these arterial branches are wide and well developed and extend over the dorsal surface of the protocerebrum. Consequently, we can hypothesize that *A. markensis* would possess a less efficient neurovascular system for oxygen supply—a plausible adaptation given the relative distance it may have from hydrothermal fluid sources compared to the two species of the *Rimicaris* genus (Desbruyères et al. 2006; Zbinden and Cambon‐Bonavita 2020). This is consistent with the hypothesis of Reiber and McMahon (1998), according to which modification of the brain perfusion efficiency is impacted by environmental factors. By being exposed longer to the fluids due to their symbiotic relationships with chemoautotrophic microorganisms, the genus *Rimicaris* (particularly *R. exoculata*) would probably need a more important amount of oxygenated hemolymph sent toward the brain than other hydrothermal vent species to compensate for potential drops in oxygen levels.

## 5 Conclusion

Despite the diverse ecological niches occupied by alvinocaridids in the MAR, the morphological characteristics of the central nervous system showed a notable degree of similarity between the four species. The similar compacted organization of the brain, the hypertrophy of the complex made up by MB and TM, and similar proportions of the other brain neuropils among the four species studied compared to the coastal *P. elegans* appear to indicate an influence of shared environmental variables such as pressure, low light, and high temperatures (Joseph 2017), rather than an influence of specific diets or ecological niches. The hypertrophy of the higher‐order neuropils, the complex comprising the MB and TM in these animals, suggests a high capacity for multimodal sensory processing.

Concerning the visual apparatus, *R. chacei* and *R. exoculata* present a much more developed retina, as well as a larger lamina. These morphological variations highlight that these organs could have an important role in these species, other than the proposed detection of near‐infrared radiation emitted by hydrothermal fluids, such as the perception of thermal radiation. Thus, the persistence of the visual system in Alvinocarididae, and more specifically the hypertrophy of the retina in what seems to be only symbiotic or mixotrophic species compared to scavengers, leads us to question this issue further. Future studies should analyze which factors might be associated with retinal hypertrophy (e.g., feeding strategies, danger avoidance), as well as the key factors that have enabled these species to retain their vision throughout their evolutionary history, and how such a singular visual system is implemented during ontogenesis in symbiotic species like *R. exoculata*.

Notable anatomical differences were observed in the myoarterial formation between the four hydrothermal vent species, with the most pronounced difference being observed in the scavenger species *A. markensis*. In this species, the associated neurovascular network appeared to be less developed with respect to, for example, the diameter of blood vessels than that of *R. exoculata* and *R. chacei*. However, the physiological and ecological implications of these modifications remain to be debated. Another interesting aspect related to the myoarterial formation is the development of muscle bundle 1, which can vary in diameter with the blood vessels. We note that this muscle bundle is well developed in *M. fortunata*, suggesting the potential for adaptive responses to higher oxygen demands caused by its metabolism, although these animals share a similar microhabitat and trophic strategies with *A. markensis*. Such variation illustrates the diversity of innovations among organisms exploiting the same environments. It would be interesting to compare this aspect from a larger ecological point of view by extending this comparison to other species of alvinocaridids to determine if there are any ecological implications in the morphology of this circulatory system (e.g., symbiotic relationships, proximity to vent fluids, etc.) that could explain such differences between species (Desbruyères et al. 2006; Lunina and Vereshchaka 2014).

Future experimental studies will need to extend the analyses presented here to compare with other alvinocaridid species (e.g., *Rimicaris kairei*, *Mirocaris indica*, etc.) in order to determine whether the cerebral organization is maintained across different alvinocaridids on a global scale or presents variation depending on various factors such as feeding strategies (symbiotics vs. scavengers), the type of environment (e.g., hydrothermal systems vs. cold seeps), or their geography. Another line of research should explore evolutionary modifications of these animals’ nervous systems through ontogeny and also between sexes. The larvae and juveniles of these species live in different environments from those of adults. A possible sexual dimorphism or ontogenetic variations in their brain anatomy may provide additional information on the biotic and abiotic factors that shape the brain and associated sensory organs in these shrimp species.

## Author Contributions

**Adrien Mathou**: observations, image reconstructions, measurements, writing – original draft. **Julia Machon**: sampling at sea, specimen preparation, image reconstructions. **Rebecca Meth**: methodology and specimen preparation, image reconstructions. **Magali Zbinden**: conceptualization, sampling at sea, writing – review and editing, supervision, funding acquisition. **Juliette Ravaux**: conceptualization, writing – review and editing, supervision. **Steffen Harzsch**: conceptualization, writing – review and editing, funding acquisition.

## Ethics Statement

No approval from an ethics committee (Declaration of Helsinki in 1995 and as revised in Brazil in 2013) was required for the design and conduct of this study.

## Conflicts of Interest

The authors declare no conflicts of interest.

## Data Availability

The data that support the findings of this study are available from the corresponding author upon reasonable request.
